# Shining the light on eating disorders, incidence, prognosis and profiling of patients in primary and secondary care: national data linkage study

**DOI:** 10.1192/bjp.2019.153

**Published:** 2020-02

**Authors:** Joanne C. Demmler, Sinead T. Brophy, Amanda Marchant, Ann John, Jacinta O. A. Tan

**Affiliations:** 1 Lecturer in Health Data Science, Swansea University, UK; 2 Professor of Public Health Informatics, Swansea University, UK; 3 Data Analyst, Swansea University, UK; 4 Professor of Public Health and Psychiatry, Swansea University, UK; 5 Associate Professor of Psychiatry, Swansea University, UK

**Keywords:** Eating disorder, anorexia nervosa, bulimia nervosa, epidemiology, incidence

## Abstract

**Background:**

Diagnosing eating disorders can be difficult and few people with the disorder receive specialist services despite the associated high morbidity and mortality.

**Aims:**

To examine the burden of eating disorders in the population in terms of incidence, comorbidities and survival.

**Method:**

We used linked electronic health records from general practitioner and hospital admissions in Wales, UK within the Secure Anonymised Information Linkage (SAIL) databank to investigate the incidence of new eating disorder diagnoses. We examined the frequency of comorbid diagnoses and prescribed medications in cases and controls in the 2 years before and 3 years after diagnosis, and performed a survival analysis.

**Results:**

A total of 15 558 people were diagnosed with eating disorders between 1990 and 2017. The incidence peaked at 24 per 100 000 people in 2003/04. People with eating disorders showed higher levels of other mental disorders (odds ratio 4.32, 95% CI 4.01–4.66) and external causes of morbidity and mortality (odds ratio 2.92, 95% CI 2.44–3.50). They had greater prescription of central nervous system drugs (odds ratio 3.15, 95% CI 2.97–3.33), gastrointestinal drugs (odds ratio 2.61, 95% CI 2.45–2.79) and dietetic drugs (odds ratio 2.42, 95% CI 2.24–2.62) before diagnosis. These excess diagnoses and prescriptions remained 3 years after diagnosis. Mortality was raised compared with controls for some eating disorders, particularly in females with anorexia nervosa.

**Conclusions:**

Incidence of diagnosed eating disorders is relatively low in the population but there is a major longer term burden in morbidity and mortality to the individual.

Eating disorders have high morbidity and the highest mortality of all mental disorders,^1^^,^^2^ yet they can be difficult to diagnose. They are characterised by disturbed eating behaviour associated with concern about weight and shape. People with eating disorders are often young and highly vulnerable yet ambivalent about recovery and may be difficult to engage with treatment.^3^^,^^4^ Without specific knowledge of the condition, people with this mental health condition can evade detection, thus delaying time to diagnosis and treatment and influencing long-term outcome. Carers for people with eating disorders experience high levels of strain.^5^ Healthcare costs for eating disorders in the National Health Service (NHS) across the UK have been recently estimated as £3.9–4.6 billion with an overall economic cost likely to be more than £6.8–8 billion per year.^6^ Over 1.6 million people in the UK are estimated to be affected by eating disorders, and this is likely to be an underestimate as many people do not seek help.^7^ In addition, it is reported that the number of people diagnosed with eating disorders has increased by 15% between 2000 and 2009,^8^ and it is not clear if this is due to increased help seeking or due to better diagnosis. Generally, across the UK there have historically been relatively scarce and variable resources for eating disorders.^9^ Given the difficulty of engaging people with eating disorders in treatment and the scarcity of resource provision for such specialist treatment, it is likely that specialist eating disorder treatment teams see a minority of those – whether child, adolescent or adult – with the disorder. General practitioners (GPs; or family physicians, in this article we will refer to them as ‘primary care’) must identify and diagnose eating disorders in patients before they can be referred to specialist services (which we call ‘secondary care’) and, in many cases and for a variety of reasons, patients may only be known to, or receive treatment in, primary care. There has been previous routine clinical databank work in the UK of incidence,^8^ and previous work on mortality of eating disorders.^10^ This is the first article in the UK to examine the burden of eating disorders including the incidence, survival and characteristics of those with eating disorders both before and after their diagnosis.

## Methods

### Study design: record-linked electronic cohort study

The linkage and hosting of data were performed in the Secure Anonymised Information Linkage (SAIL) databank.^11^^–^^15^ The SAIL databank is a large routine clinical database covering the whole of Wales. Anonymised record linkage is carried out in the SAIL databank on routinely collected data held in health and social care data sets and supported by the national institute for Health Data Research UK (HDR UK; https://www.hdruk.ac.uk). For each data set within the SAIL databank, individuals’ identities are removed and replaced with an Anonymised Linking Field (ALF), based on their names, address or NHS number, which is used to link across data sets. All data within the SAIL Gateway are treated in accordance with the Data Protection Act 2018 and is compliant with the General Data Protection Regulation. Appropriate disclosure control methods were used to restrict the reporting of small numbers (i.e. categories containing less than five individuals).

We used data from a variety of data sets between 1 July 1990 and 30 June 2017 (start and end of primary care data) which were placed in SAIL by the Welsh NHS and other data holders. The GP data covers, at the time of writing, 77% of all GP practices in Wales. The Welsh Demographics Service (WDS) contains key statistics – such as gender, week of birth and date of death – for everyone in Wales registered with a GP. The Patient Episode Database for Wales (PEDW) contains all in-patient hospital admissions to Welsh hospitals. The Office of National Statistics (ONS) data set covers all births and deaths in Wales. The ONS, WDS and PEDW data are available for the whole of Wales.

### Data preparation

We verified individuals with a diagnosis of an eating disorder using Read Version 2^16^ diagnosis codes in primary care data (visits to family physician) and ICD-10 (1992) diagnosis codes^17^ in secondary care data (hospital admissions) between the ages of 10 and 65 years. The date of first mention of eating disorder (from GP or hospital data) was used for each individual, so a diagnosis is only recorded once for each person. The use of the ALF unique to each individual ensures that individuals are not double counted when they appear in both GP and secondary care data. Only individuals with either an exact match on NHS number or demographics, or a probabilistic match of over 90% were included in this study. Eating disorders were divided into three groups, i.e. (a) anorexia nervosa, (b) bulimia nervosa and (c) other eating disorders, using a hierarchical system. The clinical codes used in Read Version 2 can be found in Supplementary Table 1, available at https://doi.org/10.1192/bjp.2019.153; those for ICD-10 are in Supplementary Table 2.

To be included in the cohort, individuals needed to have been correctly matched to the WDS (i.e. had an ALF) and have a week of birth and gender recorded. If there were multiple diagnoses codes and dates, we kept the earliest diagnosis date and the top diagnosis category (i.e. ‘anorexia nervosa’ in preference to ‘bulimia nervosa’ and ‘bulimia nervosa’ in preference to ‘other eating disorders’, which encompassed all other eating disorder diagnoses). We finally created a combined cohort of anyone with an eating disorder diagnosis in either the GP or hospital admissions data, using the same hierarchical rules.

The Read codes were validated using the methodology employed by the Health Informatics Trial Enhancement Project.^18^ This methodology has proved both secure and effective in validating mental health algorithms with the SAIL databank. Two independent mental health clinicians were given secure access to this data set to rate eligibility for inclusion in the ‘e-cohort’. This method is particularly advantageous in allowing sensitivity and specificity to be calculated by using clinical judgement as the gold standard with no risk of participant identification. A kappa statistic was obtained to measure the agreement between the two clinicians, an eating disorder psychiatrist and a GP with mental health expertise. The kappa value was 0.82, showing a high level of agreement. Analysis was conducted using SPSS version 20 for Windows.

### Analysis

Data linkage and data preparation within the SAIL databank for Windows 7 Enterprise were conducted using IBM DB2 9.7 SQL. Data were then imported into R (version 3.4.1), which was used for most statistical analyses. Survival analysis was executed in Stata 15. Results from primary and secondary care extracts were combined to create a joined eating disorder cohort.

### Incidence

The denominator of the diagnosed incidence of eating disorders in secondary care was calculated based on the total number of individuals with recorded gender, registered and living in Wales between 1 July 1990 and 30 June 2017 for each full year of the study, respectively. The primary care denominator was further adjusted to include only people living in Wales and whose GP practice was contributing data to SAIL. The incidence in primary and secondary care was then added together to create an overall estimate of the incidence in Wales. Descriptive statistics were used to examine the age and year of first diagnosis, stratified by eating disorder type. A Student's *t*-test was used to investigate the difference in the mean age of diagnosis by diagnosis type and age.

### Characteristics of patient before and after diagnosis, stratified by eating disorder type

People diagnosed with an eating disorder were matched on gender and age at diagnosis with four controls per patient with an eating disorder, using a computer algorithm. Randomisation was used if there were multiple potential matches with equal distance. Both patients and controls were required to have 2 years of GP data before (cohort 1) or 3 years of GP data after (cohort 2) diagnosis/matching date. (Results for a much smaller single case–control cohort spanning all 5 years were added for comparison in Supplementary Fig. 7.) The period of available data at each GP practice was defined as the period when the practice was recording a sufficient volume of events, and was therefore believed to be regularly coding data in an electronic system. The volume of events over time was defined as Read-coded events per person-month, and periods of time where the volume was at least 10% as high as 2009 (a known good period of data) were included. Patients with less than four controls were excluded, i.e. each patient had exactly four controls. We examined the frequency of codes in Read chapters ‘A–Z’ (diagnoses) and ‘a–z’ (prescriptions) in the 2 years before a diagnosis compared with controls, and separately in the 3 years after diagnosis. The odd ratios of a diagnosis/prescription were calculated for all Read chapters and Read sections or subsections of interest. We used this method as a means to profile anything that might be of importance to people with eating disorders instead of concentrating on codes that are known to be of importance.

### Survival analysis

Cox regression and Kaplan–Meier estimates were used to examine time to death stratified by eating disorder type, compared with controls. Data were censored if a person moved out of Wales or moved to a GP that did not return data to SAIL. Patients who move between GPs who contribute to SAIL (80% of all practices in Wales) can be linked across GP practices.

### Ethical approval

The study design uses anonymised data and therefore the need for ethical approval and participant consent was waived by the approving Institutional Review Board, UK NHS Research Ethics Committee. The SAIL independent Information Governance Review Panel (IGRP), which contains members from the UK NHS Research Ethics Committee, experts in information governance and members of the public, approved the study.

### Data sharing

The data used in this study are available in the SAIL databank at Swansea University, Swansea, UK. All proposals to use SAIL data are subject to review by an independent IGRP. Before any data can be accessed, approval must be given by the IGRP.

The IGRP gives careful consideration to each project to ensure proper and appropriate use of SAIL data. When access has been granted, it is gained through a privacy-protecting safe haven and remote access system referred to as the SAIL Gateway. SAIL has established an application process to be followed by anyone who would like to access data via SAIL: https://www.saildatabank.com/application-process.

## Results

### Eating disorders in GP data

**Fig. 1:**
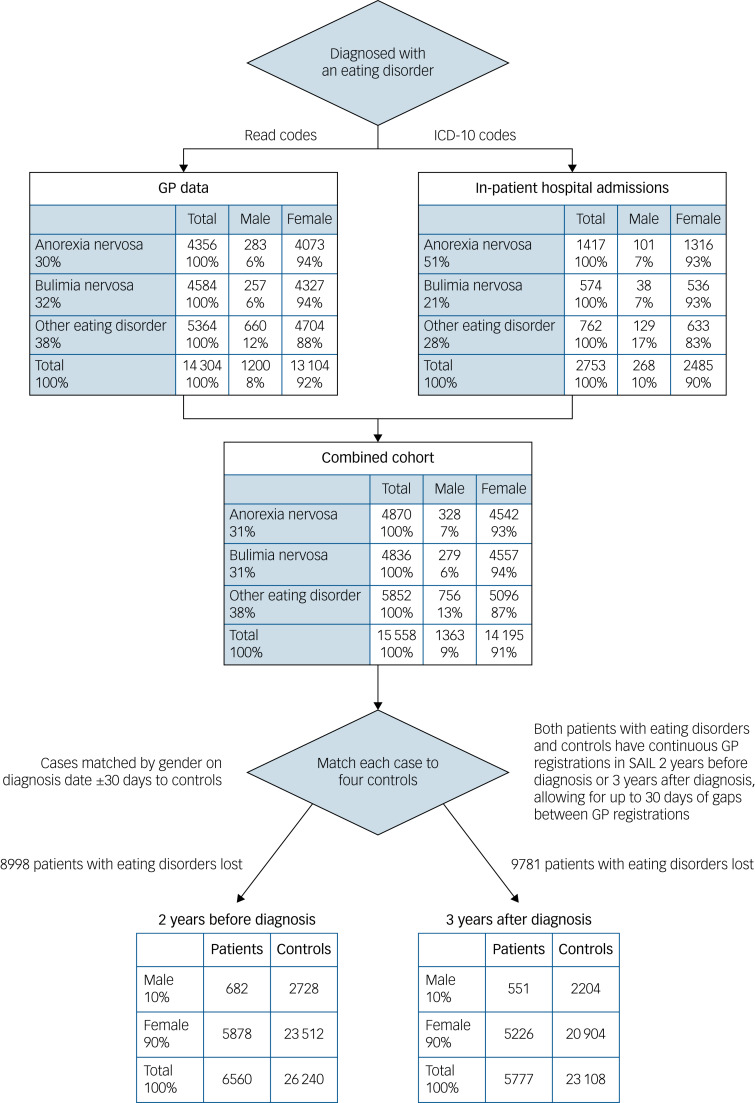
Flow chart of data preparation.

GP, general practitioner; SAIL, Secure Anonymised Information Linkage.

The GP data contained a total of 14 304 individuals aged between 10 and 65 years that had been diagnosed with an eating disorder between 1990/91 and 2016/17. Anorexia nervosa was diagnosed in 4128 individuals, bulimia nervosa in 4584 individuals and other eating disorders in 5364 individuals (see Fig. 1 GP data).

The age at first recorded diagnosis peaked in the age group 15–19 years for both genders (see Supplementary Fig. 1). The median age at diagnosis with any eating disorder was 20. Anorexia nervosa and other eating disorders were diagnosed earlier than bulimia nervosa. There was a statistically significant difference in the age of diagnosis between men and women with anorexia (male 25 years, female 22 years; 95% CI 1.22–4.25) and bulimia nervosa (male 27 years, female 25 years; 95% CI 0.78–3.45), with males being diagnosed later than females.

The mean number of new diagnoses (incidence) per GP practice in each year ranged from 1.6 to 3.0 per practice size of 11 500 patients with its peak in 2004/05. The maximum number of cases (prevalence) in a single GP practice was 26.

### Incidence over time

**Fig. 2:**
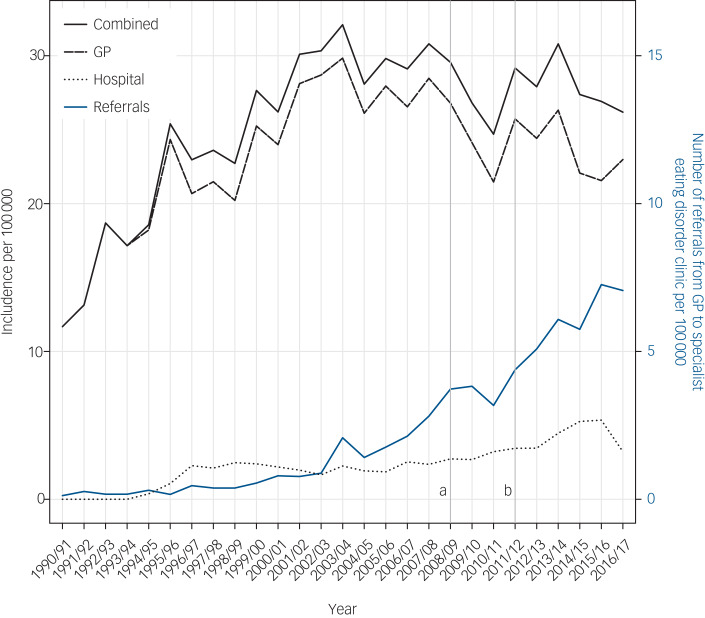
Incidence of eating disorders in the combined cohort and number of referrals to specialist eating disorders services over time. (a) Framework for eating disorder services, (b) start of specialist eating disorder services in Wales.

The incidence of diagnosed eating disorders in the Welsh population (taken from GP and hospital data) ranged from 8 per 100 000 people in 1990/91 to 24 per 100 000 people at its maximum in 2003/04 (see Fig. 2). Although coded diagnoses of eating disorders in GP practices have declined slightly since 2008, referrals by GPs to specialist eating disorders clinics has progressively increased since 2000. In addition, hospital admissions with eating disorders have also continued to rise (see Fig. 2). However, in general, the majority of eating disorders are not referred or found in hospital (see Fig. 1).

GP, general practitioner.

### Characteristics of people with eating disorders

A total of 15 558 (male 1363, female 14 195) had a diagnosis of an eating disorder in either primary or secondary health records. A total of 6560 patients with eating disorders (42.2% of the total) had continuous GP records in the SAIL databank and could be matched to 4 controls for 2 years before their eating disorder diagnosis. Similarly, 5777 patients with eating disorders (37.1% of the total) had continuous GP records in the SAIL databank and could be matched to 4 controls for 3 years after their eating disorder diagnosis (see Fig. 1). The main reason for the loss of patients were migration (i.e. people were not registered with a GP in SAIL for the whole 5-year follow-up period) and GP data coverage (i.e. years of low data volume for individual GP practices).

**Fig. 3:**
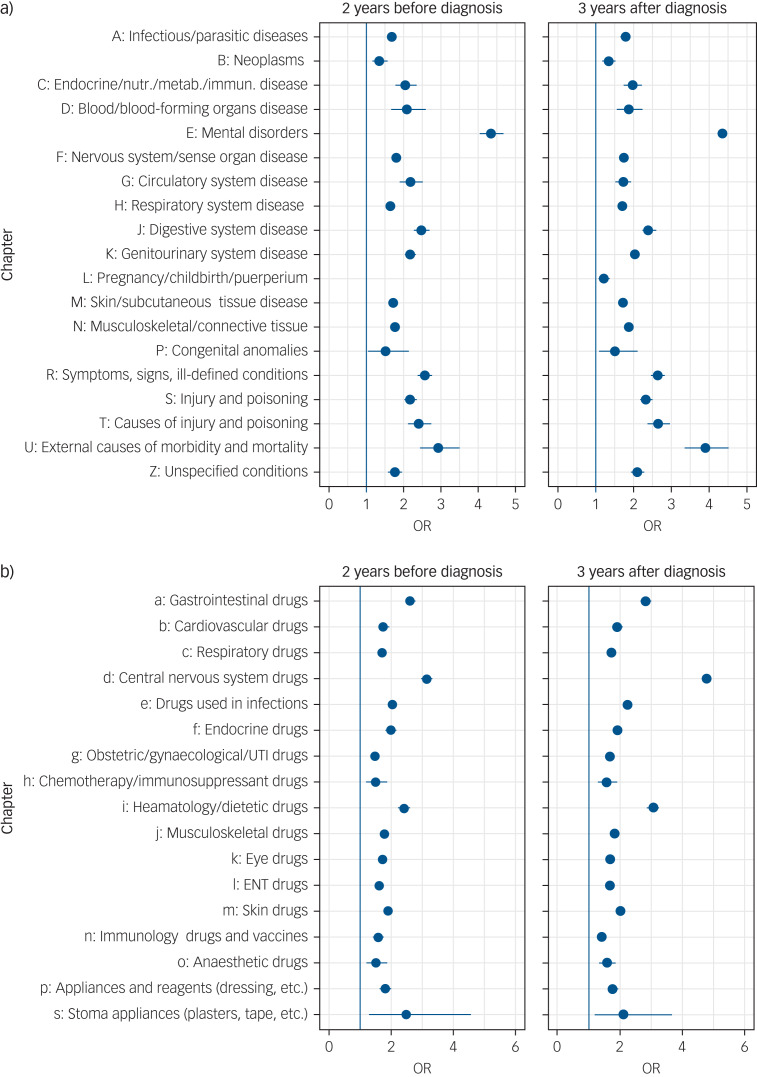
Odds ratios for (a) diagnoses and (b) prescriptions 2 years before or 3 years after an eating disorder diagnosis. Diagnoses exclude eating disorders. Only significant results at 95% confidence intervals with at least 10 affected cases or 40 affected controls are displayed.

Significant differences in diagnosis and prescription codes between patients and controls before and after diagnosis are shown in Fig. 3. Those with eating disorders showed higher levels of mental disorders (odds ratio 4.32, 95% CI 4.01–4.66) and external causes of morbidity and mortality, for instance self-harm (odds ratio 2.92, 95% CI 2.44–3.50) before their diagnosis. The largest odds ratios for mental disorders before diagnosis were found for personality disorders (odds ratio 10.82, 95% CI 6.58–18.56), alcohol dependence syndrome (odds ratio 6.03, 95% CI 3.92–9.40) and depressive disorders (odds ratio 5.93, 95% CI 4.78–7.37). All statistically significant mental health results can be found in Supplementary Fig. 2.

Endocrine/nutr./metab./immun. disease, Endocrine, nutritional, metabolic and immunity disorders; OR, odds ratio; UTI, urinary tract infection; ENT, ear, nose and throat.

Central nervous system drugs (including psychiatric drugs) (odds ratio 3.15, 95% CI 2.97–3.33), gastrointestinal drugs (odds ratio 2.61, 95% CI 2.45–2.79) and dietetic drugs (odds ratio 2.42, 95% CI 2.24–2.62) were more often prescribed in the patients with eating disorders in the 2 years before their diagnosis then in the controls. Prescription levels differ between young people and adults (see Supplementary Fig. 6).

The most relevant central nervous system drugs prescribed before diagnosis where antipsychotics (odds ratio 6.78, 95% CI 5.48–8.40), tricyclic antidepressants (odds ratio 3.73, 95% CI 3.28–4.24), other antidepressants (odds ratio 5.24, 95% CI 4.87–5.64), hypnotics (odds ratio 4.31, 95% CI 3.79–4.90) and anxiolytics (odds ratio 4.05, 95% CI 3.58 anxiolytics 4.59) (see also Supplementary Fig. 3).

People with eating disorders also showed a high amount of gastrointestinal drug prescriptions (see Supplementary Fig. 4) and exhibited much higher levels of dietetic supplement prescriptions (odds ratio 19.01, 95% CI 14.59–25.16), multivitamin preparations (odds ratio 8.37, 95% CI 5.82–12.22) and other preparations (see Supplementary Fig. 5).

### Survival

Deaths occurred across a wide time span from when the eating disorder diagnosis was first made within SAIL, with 6% of patients with anorexia nervosa dying within 15 years of diagnosis. Patients with anorexia nervosa were significantly more likely to die than their controls (hazard ratio 2.33, 95% CI 1.95–2.78). This was particularly evident for females with anorexia nervosa (hazard ratio 2.53, 95% CI 2.09–3.05). Slightly higher mortality was also found in bulimia nervosa (hazard ratio 1.41, 95% CI 1.13–1.779) and in other eating disorders (hazard ratio 1.82, 95% CI 1.53–2.18), see also Table 1.

**Table 1:** Time to death or end of follow-up hazard ratios for case–control cohort

	Number of deaths patients and controls	Follow-up years	Crude incidence rate (95% CI)	Crude hazard ratio (95% CI)
Anorexia nervosa				
Total (*n* = 24 348)	525	320 473	0.164 (0.150–0.178)	2.33 (1.95–2.78)
Male (*n* = 1640)	73	19 795	0.369 (0.293–0.464)	1.31 (0.77–2.23)
Female (*n* = 22 708)	452	300 678	0.150 (0.137–0.165)	2.53 (2.09–3.05)
Bulimia nervosa				
Total (*n* = 24 174)	390	333 855	0.117 (0.106–0.129)	1.41 (1.13–1.77)
Male (*n* = 1395)	60	16 000	0.375 (0.291–0.483)	1.45 (0.82–2.58)
Female (*n* = 22 779)	330	317 856	0.104 (0.093–0.116)	1.41 (1.10–1.80)
Other eating disorders				
Total (*n* = 29 255)	570	29 255	0.183 (0.168–0.198)	1.82 (1.53–2.18)
Male (*n* = 3 775)	160	3775	0.439 (0.376–0.512)	1.63 (1.16–2.30)
Female (*n* = 25 480)	410	25 480	0.149 (0.135–0.164)	1.91 (1.55–2.35)
Total (*n* = 77 777)	1485	966 606	0.154 (0.146–0.162)	1.87 (1.68–2.09)
Male (*n* = 6810)	293	72 261	0.405 (0.362–0.455)	1.51 (1.17–1.95)
Female (*n* = 70 967)	1192	894 345	0.133 (0.126–0.141)	1.97 (1.74–2.22)

The leading type of cause of death in the eating disorder cohort was diseases of the respiratory system (17.9% of deaths; includes bronchopneumonia, pneumonia, chronic obstructive pulmonary disease, pneumonitis due to food and vomit, and respiratory failure), followed by injury, poisoning and certain other consequences of external causes (13.3% of deaths; includes poisoning, asphyxiation) and diseases of the digestive system (11.4% of deaths; includes gastrointestinal haemorrhage, alcohol related, hepatic failure, hepatorenal syndrome, cirrhosis of liver). In the eating disorder cohort, 82% of deaths had an assigned cause of death. In contrast, the leading cause of death for the control cohort was neoplasms (17.9% of deaths), followed by diseases of the circulatory system (10.1%; includes heart diseases, stroke) and diseases of the respiratory system (8.0% of deaths; includes bronchopneumonia, pneumonia and respiratory failure). However, only 58% of the deaths in the control cohort had an assigned cause of death.

## Discussion

Historically in Wales there has been a paucity of adult eating disorder services, with specialist services specifically for the treatment of adult eating disorders being systematically set up across the country in 2011.^19^ Owing to the tier service model used, these specialist services only accept the most severe and/or medically complex cases of eating disorders among adults. Less severe cases of eating disorders among adults and most children and adolescents with eating disorders are seen in community general mental health services. The study finds the majority of eating disorder cases are seen by GPs and, despite the low incidence, they may require more resources and expertise to treat the disorder successfully. An average GP practice will see between one and three new cases of eating disorders in young people every year, and these patients will show higher rates of presentation with gastrointestinal, dietetic and mental health disorders (personality disorders, alcohol dependence syndrome and depressive disorders) and these conditions both precede and continue for at least 3 years after diagnosis. The death rate is highest for those with anorexia nervosa at approximately 1.7 per 1000 person-years at follow-up (see Table 1). However, this is far less than previously published figures of 5.1 deaths per 1000 person-years.^10^ As an observational study, we are unable to speculate about why the overall mortality is much lower than reported elsewhere, nor of the reasoning behind the primary causes of death that are reported in the death certificates. In addition, in recent years we find the incidence of new diagnoses of eating disorders is levelling off or showing a trend to declining. This is largely consistent with a recent epidemiological study in the Netherlands.^20^

The incidence rate found in our study of 24 per 100 000 people in 2003/04 compares with a recent article from England, using the Clinical Practice Research Datalink with 400 GP practices, which found an overall crude incidence rate of 33 (95% CI 30.7–35.3) per 100 000 in 2000 and 36 (95% CI 34.4–39.2) per 100 000 in 2009. There appears to be a levelling off/ decrease in diagnoses in the GP data, although this is not reflecting referral trends in eating disorder specialist services. This finding of no increase in diagnoses at the GP level is consistent with recent reports that the population incidence of bulimia nervosa appears to be decreasing and the incidence of anorexia nervosa is plateauing.^20^^,^^21^ The increasing eating disorder referrals from GP in the data echo anecdotal reports from across the UK that referrals for both adult and child and adolescent specialist eating disorder services are increasing year upon year. The increasing referral rates may reflect increasing awareness among GP of eating disorders, alongside awareness of expansion of specialist eating disorder services across Wales and the rest of the UK.

The identification of those with eating disorders is often difficult and delayed.^22^ The increased prescription of central nervous system drugs (which includes psychiatric medication) and dietetic drugs in the 2 years before first diagnosis of an eating disorder suggests that these individuals were already presenting to their GPs in this time with weight loss and psychiatric symptoms well before first diagnosis. Other diagnoses of anxiety, neurotic disorders, acute reaction to stress and depressive disorder which were found in significantly more patients with eating disorders in our data sets, are also common comorbidities with eating disorders.^23^

There are changes pre- and post-diagnosis in increased prescription for antipsychotic medication, increased prescription for antidepressant medication and increase in nondependent misuse of drugs. Antipsychotics have been reported to play an important role in managing eating disorder,^24^ and antidepressants were found to be prescribed to nearby a third of patients with eating disorders in another study.^25^ Alcohol^26^ and drug misuse^27^ have previously been reported as frequent comorbidities in eating disorders. Alcohol misuse has also been shown to be an important predictor of mortality in anorexia nervosa.^28^^,^^29^

### Limitations

Using a hierarchical diagnosis system for the whole study period means that we can only make statements about the top eating disorder category for people within the time period. This study only reports on diagnosed eating disorders that are both known to the healthcare practitioners and coded into patient records within the study period; as a result we cannot estimate or report on undiagnosed or pre-existing eating disorders, and so incidences are only for clinical presentation and will always be expected to be an underestimate of the true numbers. Furthermore, it is not possible to report on the prevalence of eating disorders in the population as the database does not enable us to determine recovery from a previously diagnosed eating disorder. The number of cases in the case–control study was only 42% of the total cohort for 2 years before diagnosis and 37% of the total for 3 years after diagnosis. The loss of cases is mainly attributed to migration during the follow-up period or due to limited data submission by individual GP practices. Given the peak age of presentation of an eating disorder diagnosis coincides with when most students go to college or university, this may reflect movement of patients who subsequently go to universities outside Wales or patients who come into Wales for study and then return home.

### Implications and future directions

This study suggests that although the incidence of eating disorders is relatively low (24 per 100 000 people per year) compared with some other mental disorders, patients with eating disorders have a significantly higher burden of morbidity and other mental health problems and thus form a considerable burden to the NHS. Furthermore, they have significantly higher prescriptions of medications for gastrointestinal, dietetic and psychiatric conditions. This illustrates the multi-system, long-term burden of both eating disorders and their physical and mental sequelae, as well as their comorbidities. This underlines the need for healthcare professionals who treat eating disorders to be prompt and skilled in their management in the community, a point stressed by recent National Institute for Health and Care Excellence guidance for eating disorders.^30^

Our findings suggest that increased diagnoses and prescriptions begin at least 3 years before a diagnosis of an eating disorder is made. Better identification and early interventions would greatly reduce the multiple burdens of eating disorders in future through better identification and early intervention.

## Supplementary material

For supplementary material accompanying this paper visit https://doi.org/10.1192/bjp.2019.153. click here to view supplementary material
