# Supplementary material for: Shining the light on eating disorders, incidence, prognosis and profiling of patients in primary and secondary care: national data linkage study
Source: Br J Psychiatry. 2019 Jul 1;216(2):105–12. doi: 10.1192/bjp.2019.153 (PMC7557634; doi:10.1192/bjp.2019.153)
Supplement: Supplementary file 1 [file S0007125019001533sup001.zip › S0007125019001533sup008.docx]

| **Category** | **Read Version 2 code** | **Description** |
| --- | --- | --- |
| A) Anorexia nervosa | 1467. | Anorexia nervosa |
|  | E271. | Anorexia nervosa |
|  | Eu500 | Anorexia nervosa |
|  | Eu501 | Atypical anorexia nervosa |
| B) Bulimia | E2751 | Bulimia (non-organic overeating) |
|  | Eu502 | Bulimia nervosa |
|  | Eu503 | Atypical bulimia nervosa |
| C) Other eating disorders | E275. | Other and unspecified non-organic eating disorders |
|  | E2750 | Unspecified non-organic eating disorder |
|  | E275y | Other specifies non-organic eating disorder |
|  | E275z | Non-organic eating disorder NOS |
|  | Eu50. | Eating disorders |
|  | Eu50z | Eating disorders, unspecified |
|  | Eu50y | Other eating disorders |
