# Supplementary material for: Shining the light on eating disorders, incidence, prognosis and profiling of patients in primary and secondary care: national data linkage study
Source: Br J Psychiatry. 2019 Jul 1;216(2):105–12. doi: 10.1192/bjp.2019.153 (PMC7557634; doi:10.1192/bjp.2019.153)
Supplement: Supplementary file 1 [file S0007125019001533sup001.zip › S0007125019001533sup009.docx]

| **Category** | **ICD-10 codes** | **Description** |
| --- | --- | --- |
| A) Anorexia nervosa | F500 | Anorexia nervosa |
|  | F501 | Atypical anorexia nervosa |
| B) Bulimia | F502 | Bulimia nervosa |
|  | F503 | Atypical bulimia nervosa |
| C) Other eating disorders | F509 | Eating disorder, unspecified |
|  | F982 | Feeding disorder of infancy and childhood |
