# Supplementary figures and images for: Shining the light on eating disorders, incidence, prognosis and profiling of patients in primary and secondary care: national data linkage study
Source: Br J Psychiatry. 2019 Jul 1;216(2):105–12. doi: 10.1192/bjp.2019.153 (PMC7557634; doi:10.1192/bjp.2019.153)

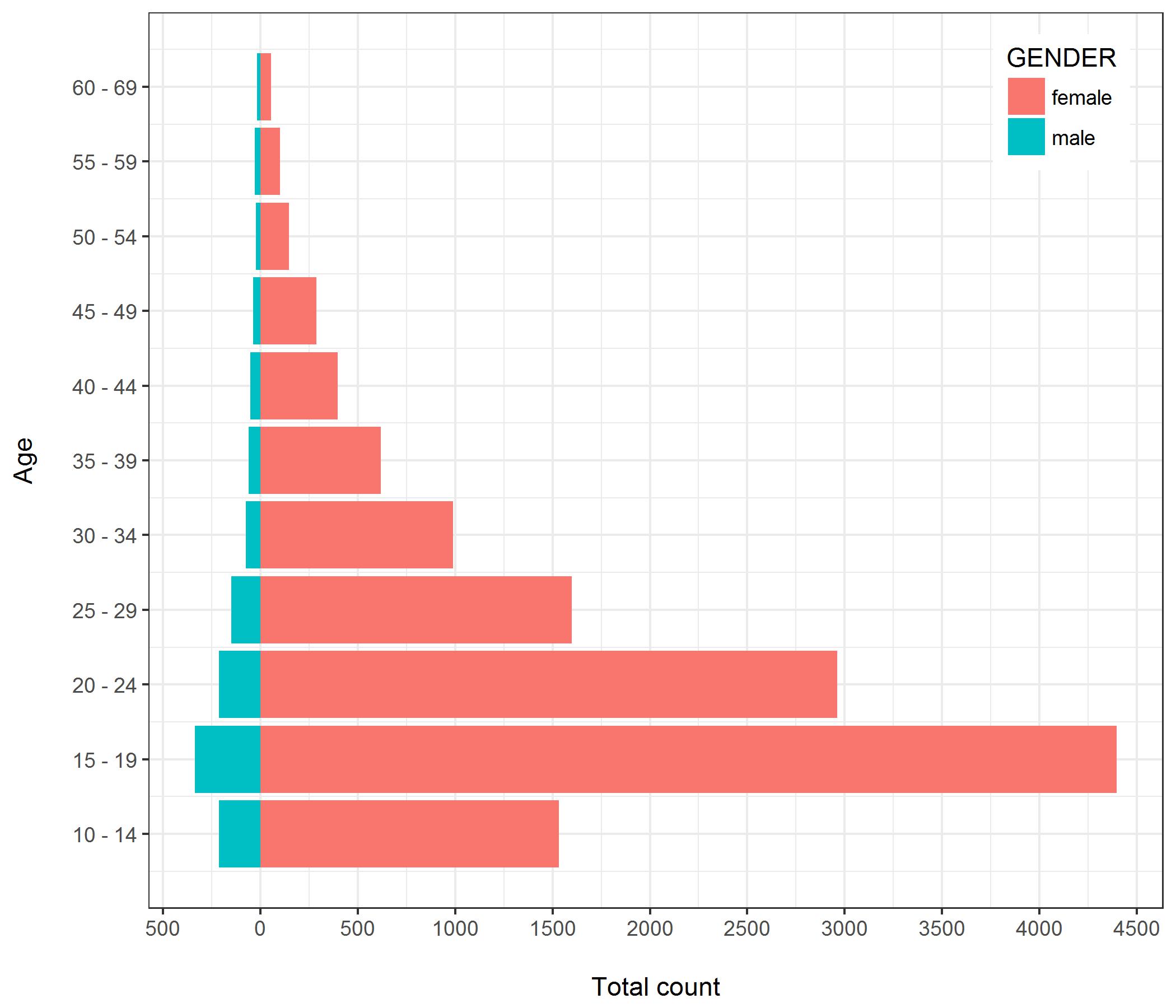

Supplement: Supplementary file 1 [file S0007125019001533sup001.zip › S0007125019001533sup001.jpg]

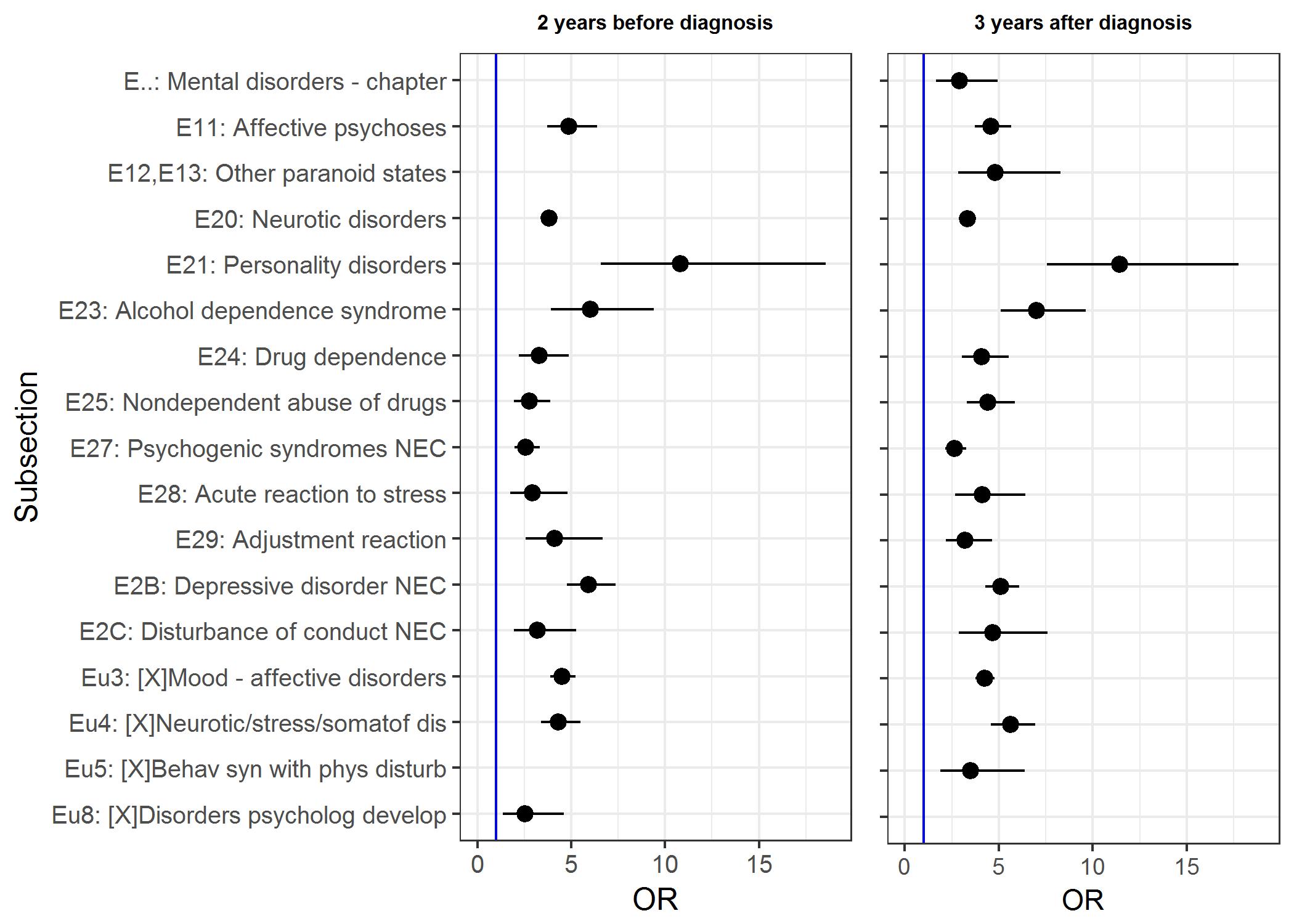

Supplement: Supplementary file 1 [file S0007125019001533sup001.zip › S0007125019001533sup002.jpg]

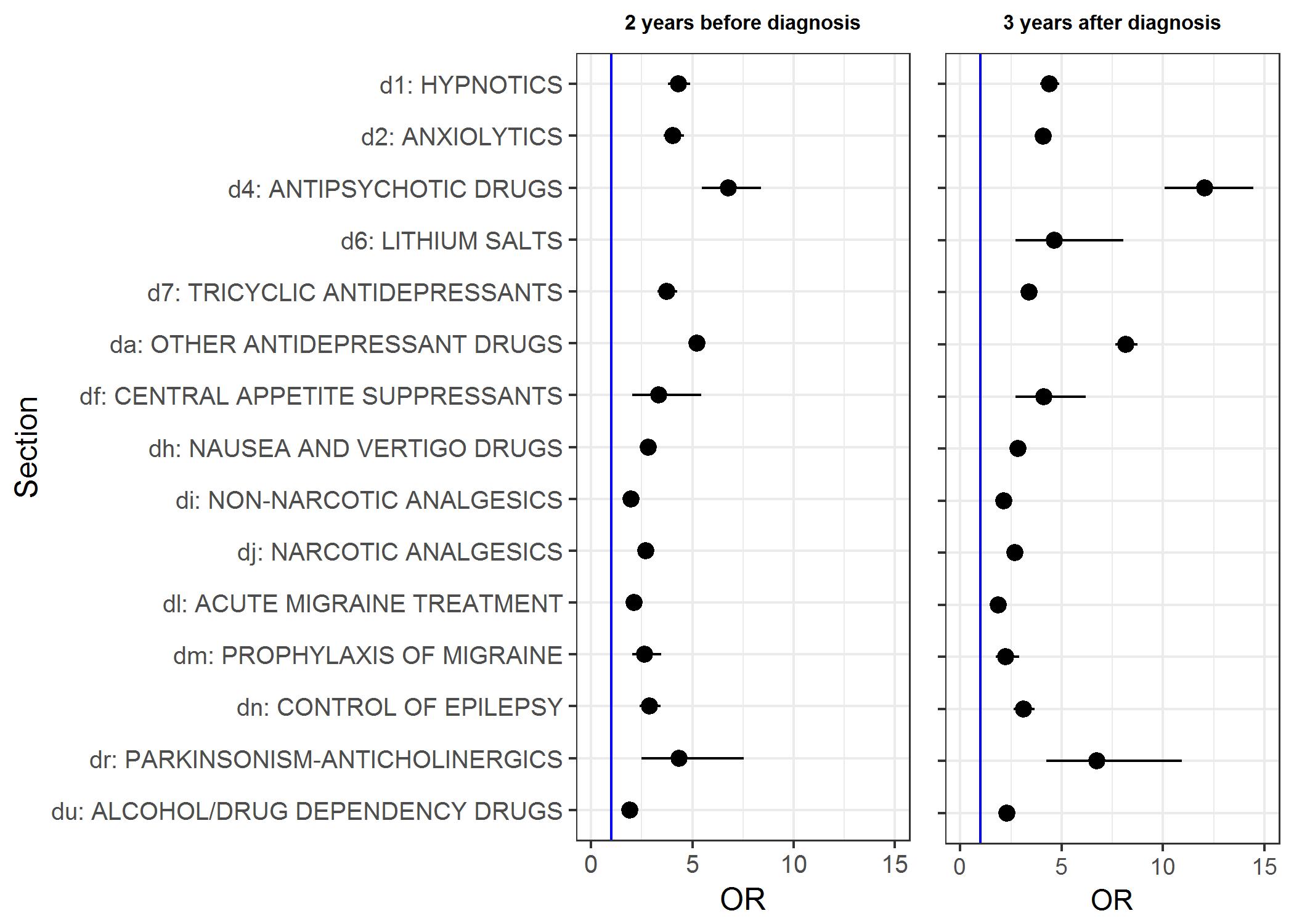

Supplement: Supplementary file 1 [file S0007125019001533sup001.zip › S0007125019001533sup003.jpg]

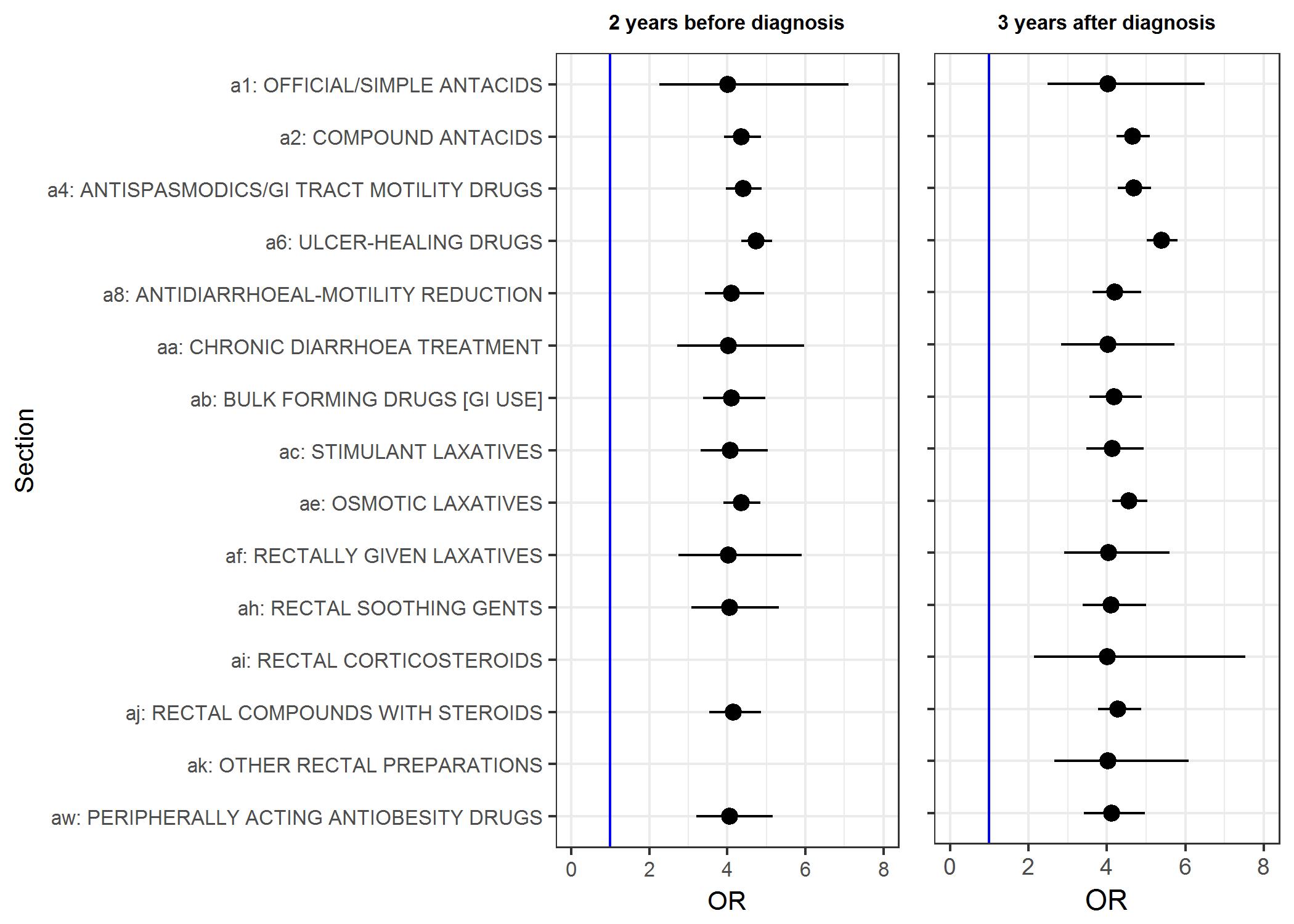

Supplement: Supplementary file 1 [file S0007125019001533sup001.zip › S0007125019001533sup004.jpg]

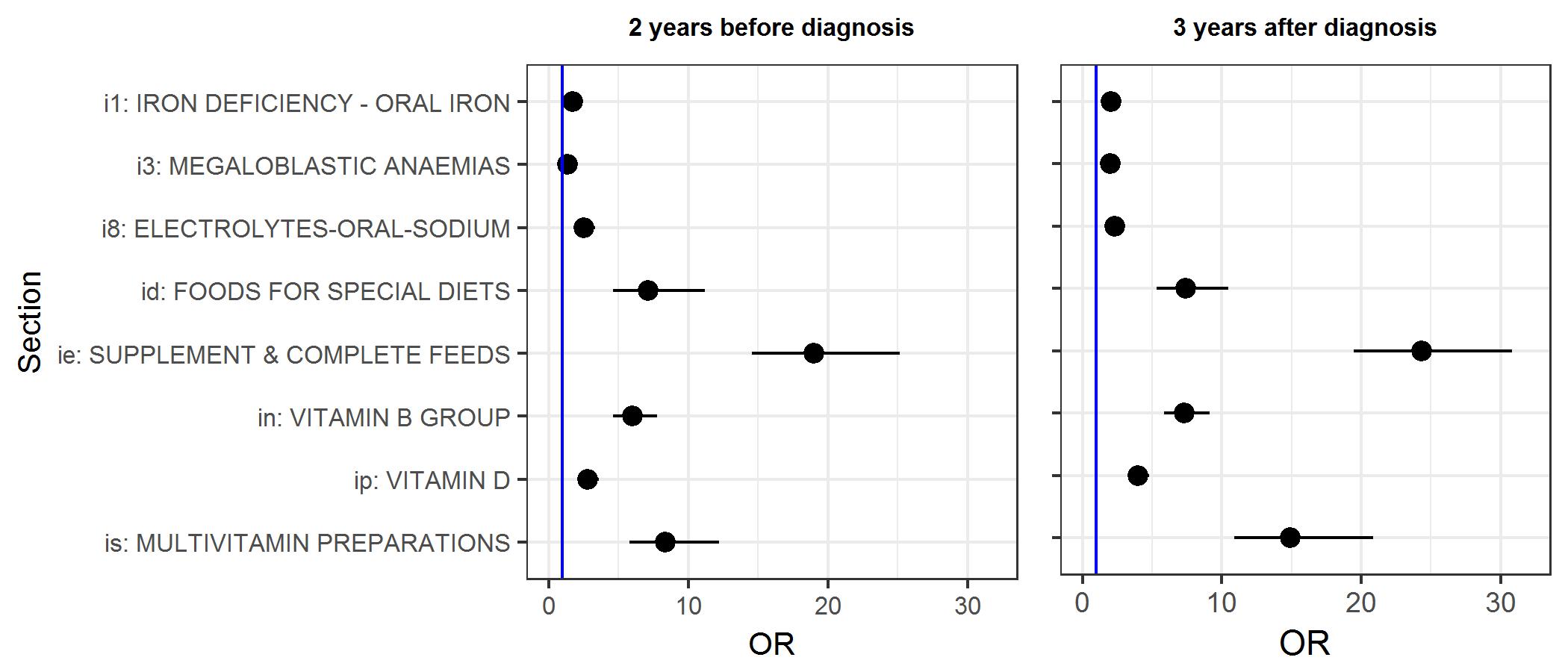

Supplement: Supplementary file 1 [file S0007125019001533sup001.zip › S0007125019001533sup005.jpg]

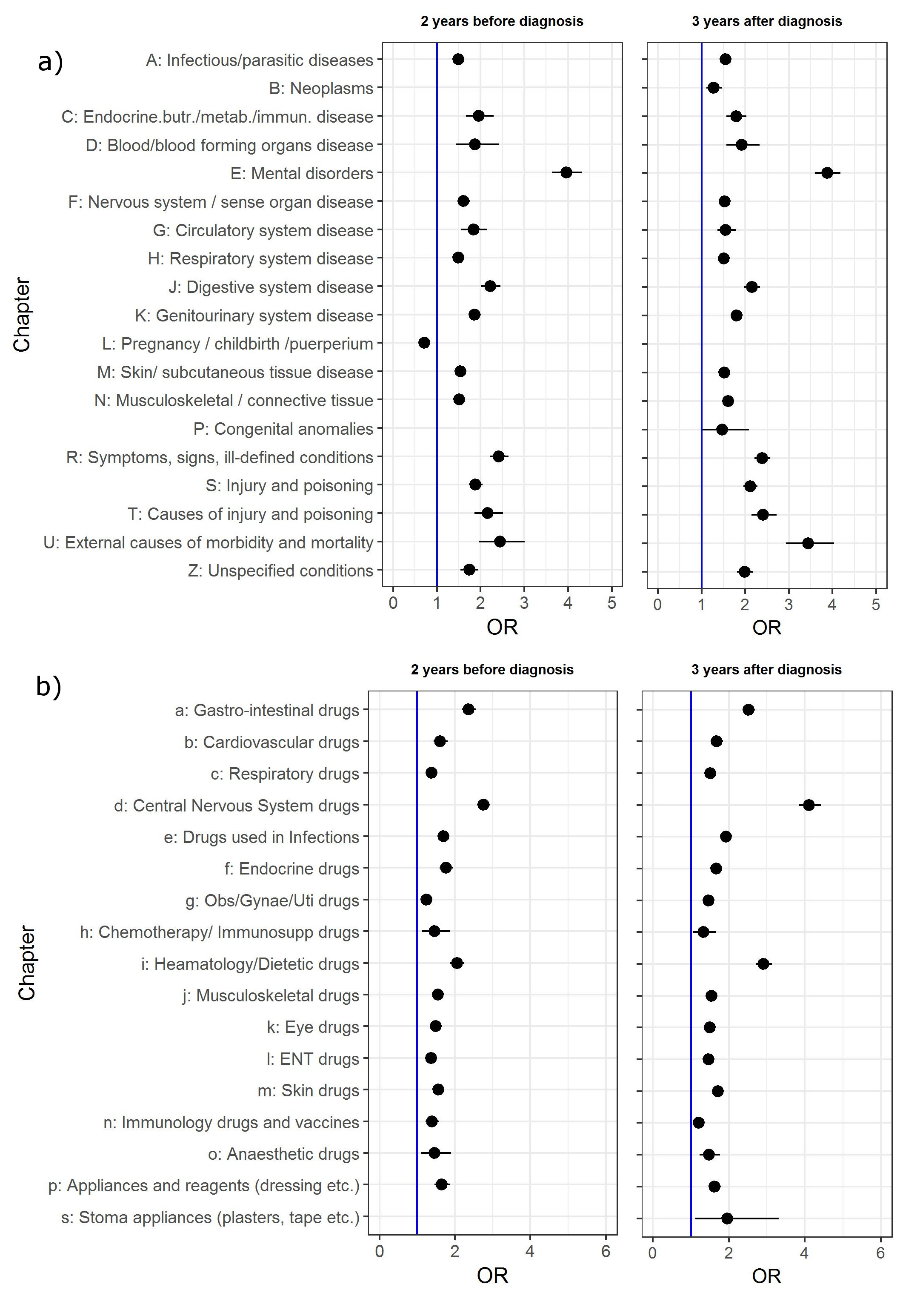

Supplement: Supplementary file 1 [file S0007125019001533sup001.zip › S0007125019001533sup006.jpg]

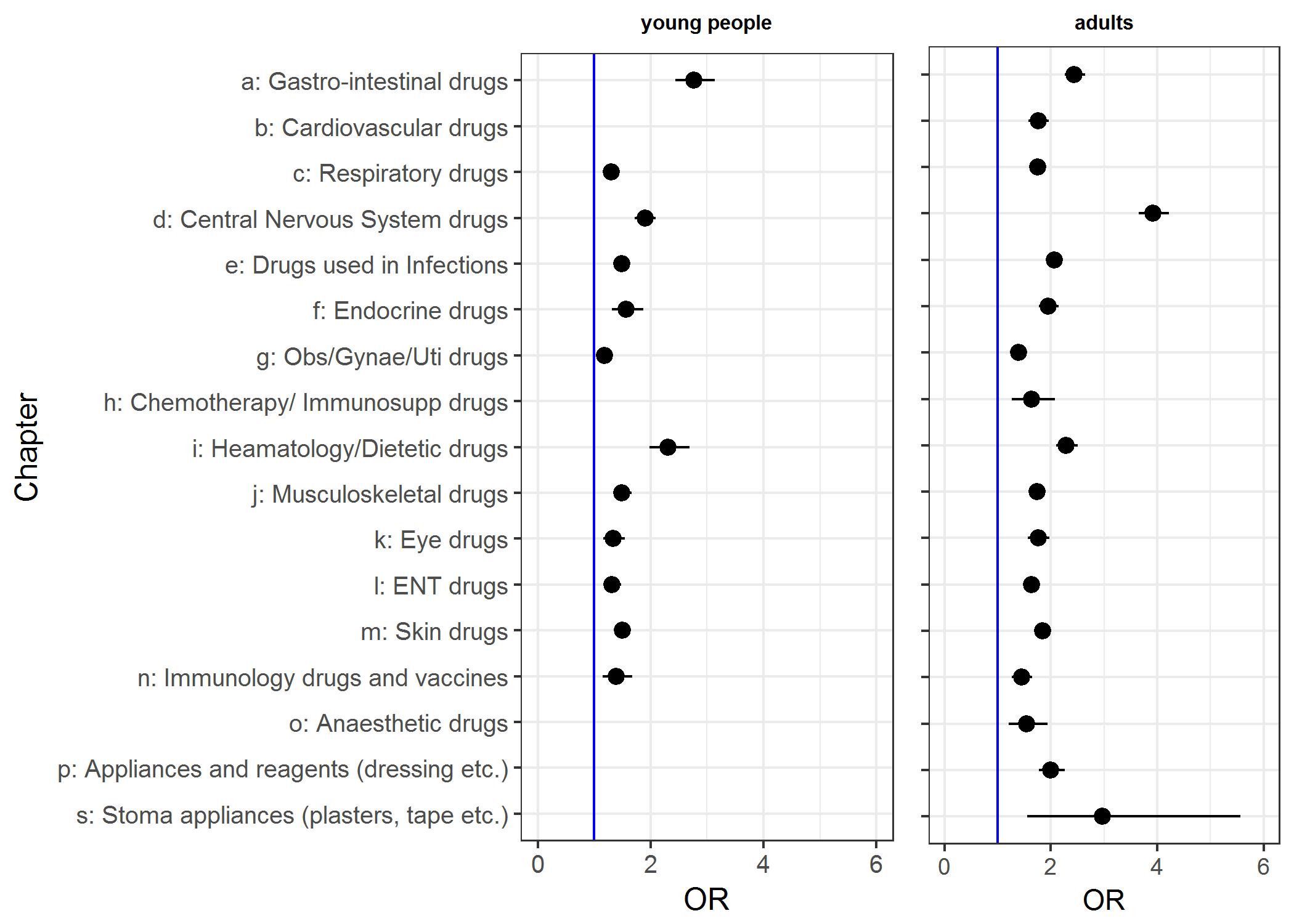

Supplement: Supplementary file 1 [file S0007125019001533sup001.zip › S0007125019001533sup007.jpg]
